# Supplementary material for: Treatment of Equine Tarsus Long Medial Collateral Ligament Desmitis with Allogenic Synovial Membrane Mesenchymal Stem/Stromal Cells Enhanced by Umbilical Cord Mesenchymal Stem/Stromal Cell-Derived Conditioned Medium: Proof of Concept
Source: Animals (Basel). 2024 Jan 24;14(3):370. doi: 10.3390/ani14030370 (PMC10854557; doi:10.3390/ani14030370)
Supplement: Supplementary file 1 [file animals-14-00370-s001.zip › animals-2759399-supplementary.pdf]

SUPPLEMENTARY MATERIALS

Table S1. Antibodies reagents used in eUC-MSC immunocytochemistry: marker, type, supplier, dilution, incubation period, positive control and cells of interest.

Marker	Type/Clone	Supplier	Dilution / Incubation period	Antigen unmasking	Positive control	Cells of interest
Vimentin	Clone V9	Dako Denmark	1/500 ON	RS/WB	Canine mammary gland	Mesenchymal/stromal cells
Pan-cytokeratin	Cocktail AE1/AE3	Thermo Scientific	1/300 ON	RS/WB	Canine mammary gland	Epithelial cells
CD31	Clone JC70A	Dako Denmark	1/50 ON	Pepsine	Canine spleen	Endothelial cells

Table S2. Average concentration of IL-6 and IL-8 in eUC-MSC CM. Concentrations obtained with Multiplexing LASER Bead analysis (Eve Technologies, Calgary, AB, Canada). The average concentration for each interleukin in eUC-MSC was evaluated in triplicate. (nd – not determined)

eUC	IL-6 (pg/mL)	IL-8 (pg/mL)
Sample 1	29.78667	13.15667
	25.03667	13.02667
	34.16667	15.95667
Sample 2	nd	nd
